# Biodiversity conservation in an anthropized landscape: Trees, not patch size drive, bird community composition in a low-input agro-ecosystem

**DOI:** 10.1371/journal.pone.0179438

**Published:** 2017-07-07

**Authors:** Eric Mellink, Mónica E. Riojas-López, Melinda Cárdenas-García

**Affiliations:** 1 Departamento de Biología de la Conservación, Centro de Investigación Científica y de Educación Superior de Ensenada, Ensenada, B.C., México; 2 Departamento de Ecología, Centro Universitario de Ciencias Biológicas y Agropecuarias, Universidad de Guadalajara, Zapopan, Jalisco, México; Justus Liebig Universitat Giessen, GERMANY

## Abstract

One of the most typical agro-ecosystems in the Llanos de Ojuelos, a semi-arid region of central Mexico, is that of fruit-production orchards of nopales (prickly pear cacti). This perennial habitat with complex vertical structure provides refuge and food for at least 112 species of birds throughout the year. Nopal orchards vary in their internal structure, size and shrub/tree composition, yet these factors have unknown effects on the animals that use them. To further understand the conservation potential of this agro-ecosystem, we evaluated the effects of patch-size and the presence of trees on bird community composition, as well as several habitat variables, through an information-theoretical modelling approach. Community composition was obtained through a year of census transects in 12 orchards. The presence of trees in the orchards was the major driver of bird communities followed by seasonality; bird communities are independent of patch size, except for small orchard patches that benefit black-chin sparrows, which are considered a sensitive species. At least 55 species of six trophic guilds (insectivores, granivores, carnivores, nectivores, omnivores, and frugivores) used the orchards. Orchards provide adequate habitat and food resources for several sensitive species of resident and migratory sparrows. The attributes that make orchards important for birds: trees, shrubs, herb seeds, and open patches can be managed to maintain native biodiversity in highly anthropized regions with an urgent need to find convergence between production and biological conservation.

## Introduction

During the last 100 years, worldwide human land use has led to natural landscape fragmentation on a considerable scale, and at a considerable speed. These changes represent major threats to biodiversity and the ecosystem services that depend upon it, and are reflected in wildlife population reductions and extinctions [1].

The need for conserving our biodiversity under such widespread human impact has been addressed through different approaches, the most popular being the establishment of protected areas. These areas were once thought to be adequate to offset biodiversity loss, but are now recognized as lacking the capacity to conserve healthy and viable populations of all life forms in many regions [1–2]. Recently, it has become clear that for successful biodiversity conservation and associated ecosystem services, anthropogenic habitats, especially agro-ecosystems, must be incorporated into conservation strategies [1, 3–5]. Indeed, in some European countries cultivated areas play a fundamental role in maintaining bird diversity and abundance [6–7].

Under an anticipated agricultural expansion and intensification [8], there is a current proliferation of research and thought on the attributes that make croplands and agro-landscapes useful for biological conservation [4, 9–12]. However, the outcome is still short-sighted, as most recent discussions and research on conservation in agricultural landscapes focus on annual, intensive, temperate croplands [2, 13–14], with some tropical systems following in a modest second place (v.gr. [10, 15]). Although it has been advocated that "conservation must identify valuable farmed landscapes and seek new mechanisms to maintain or mimic important land-management techniques in developing countries" [16], agro-ecosystems have been poorly studied for their conservation potential.

In arid and semi-arid regions, some traditional oasis-based and rain-fed agro-ecosystems support high bird species richness and play a role in biological conservation [12, 17–18]. In these regions, and in other parts of the world, perennial agro-ecosystems, especially those under low-input, dry-farmed schemes, may be useful for biodiversity conservation. Several candidate crops of this type exist: nopal (*Opuntia* spp., prickly pear cacti), olives, carob, pomegranates, almonds, figs, and pistachios. For example, traditional, rain-fed olive groves around the Mediterranean are an important habitat for wild animals, including both resident and migratory birds [19–21], although the assessments of the attributes that can enhance or endanger biodiversity conservation have not been adequately assessed.

One common agricultural system in Mexico’s southern portions of the altiplano (higland), is that of fruit-oriented nopal orchards. Although focused on the production of tunas (the pulpy fruits of nopales) for market consumption, orchards are also a source of goods for self-consumption (tunas, tender cactus pads, an occasional edible white-tooted packrat [*Neotoma leucodon*], or a cottontail rabbit [*Sylvilagus audubonii*], and as emergency fodder), in addition to providing habitat suitable for wildlife. Thus, these orchards are valuable for biological conservation [3, 12, 22].

Nopal orchards are a peculiar form of agrohabitat, that conforms rather well to the concept of "more-natural" habitats of Fahring et al. [14], as they comply with the statement "most primary production is not consumed by humans… the main species have an evolutionary or long-term association with [the cover type]… and… the frequency and intensity of anthropogenic disturbances are low". This makes them, in addition to their potential value for conservation as part of an eco-agricultural approach (*sensu* [5]), a good model to study the attributes that can be tailored to improve the value of perennial fruit-producing agro-ecosystems for biological conservation.

Fruit-production nopal plantations do not need to be taken out of production in order to provide suitable habitat for a large number of wild vertebrate species [3, 12, 23–25]. In our study region in central Mexico [23], at least 9 reptiles, 26 birds, and 54 mammals consume tunas, flowers, and pads, and many more use the orchards as habitat, including at least 112 bird species. In addition, nopal plants effectively retain soil and rainwater [26].

With more than 55 000 ha planted in the country, cultivated tunas are the 10th most important perennial fruit crop in Mexico, and third among the crops originally from the Americas (following avocado and cacao; [27]). In our study area there are >20 000 ha of this crop [27]. Due to its hardiness, nopal orchards have been established in several countries in the Mediterranean region, northern Africa, Middle East, South Africa, South America, and North America [26, 28]. Thus, knowledge on how to tailor nopal orchards to be more valuable for wildlife has the potential to have a large conservation impact, as the same attributes that drive ecological processes in orchards in one area would presumably operate throughout the world.

This agro-ecosystem is subject to the same ecological drivers that operate in natural and semi-natural semi-arid habitats. Habitat heterogeneity, the presence of arboreal elements, and other “keystone structures” such as live fencerows and rock fences, and open herb patches are among the major attributes that influence the value of a habitat for wildlife [7, 4, 29]. Recently we documented that in the highly anthropized Llanos de Ojuelos, Mexico, nopal orchards had more bird species than two other widespread regional habitats, grasslands and annual rain-fed croplands, and did not differ from natural shrublands [12]. Being low-technology agro-ecosystems, nopal orchards vary in their internal structure, size and shrub/tree composition according to individual farmers' decisions, and economics [3, 12].

Among the habitat attributes that have been touted as primarily important, patch size [30–31], and vertical habitat heterogeneity [13, 29] stand out for the recurrence and magnitude of their impact on wild birds. However, despite the general conception that large patches are better for biological conservation, small patches can be very valuable to this end, but this depends strongly on their internal structure and degree of connectivity [32].

The Llanos de Ojuelos has been subject to extensive landscape changes, but the region retains most or all faunal species that can be expected there, according to species distribution maps in the literature. However, conservation of all local species is not warranted as natural and semi-natural secondary habitats continue to be transformed, through both plowing and grazing. No protected areas exist in the region, and human population density and needs make the creation of one that is large enough to ensure the maintenance of species and processes very unlikely. As an alternative, we have demonstrated that nopal orchards can be tailored to meet specific conservation needs, although many processes in them remain to be better understood. To aid in understanding further the conservation potential of nopal orchards, we conducted a study aimed at evaluating the effect of patch-size and the presence of trees in them on bird community composition.

This study was focused on unraveling those orchards´ characteristics that are more critical to the conservation of biodiversity, especially in that of birds, within a landscape context. The goal was to provide decision makers with management actions to help reconcile agricultural production with biological conservation.

## Material and methods

### Study area and sites

The Llanos de Ojuelos, in the southern part of Mexico´s Altiplano, is an extended semi-arid plateau 1800–2300 m above sea level, with a mean annual temperature of 17°C, and 473.5 mm of rain, which falls almost all during the summer (see Fig 1 in [12]). This area is strongly anthropized by processes that began with the arrival of Spanish colonizers in the XVI century [3].

The original landscape of rolling hills covered by large arboreal nopaleras (prickly pear communities with nopales, *Opuntia* spp., up to 6–7 m tall), grasslands, and some shrublands, with oak forests on the mesas, ravines, and flanks, has been transformed into one dominated by rain-fed agricultural fields and overgrazed grasslands. The are often interspersed with shrublands, many of which are the result of overgrazing [3, 11, 22, 24], and some nopal-shrub-grass communities resulting from soil conservation programs. Of the original dense arboreal nopaleras, only small patches remain, while oak forests seem to be recovering as a result of use of domestic firewood having been replaced by butane gas stoves.

**Fig 1:**
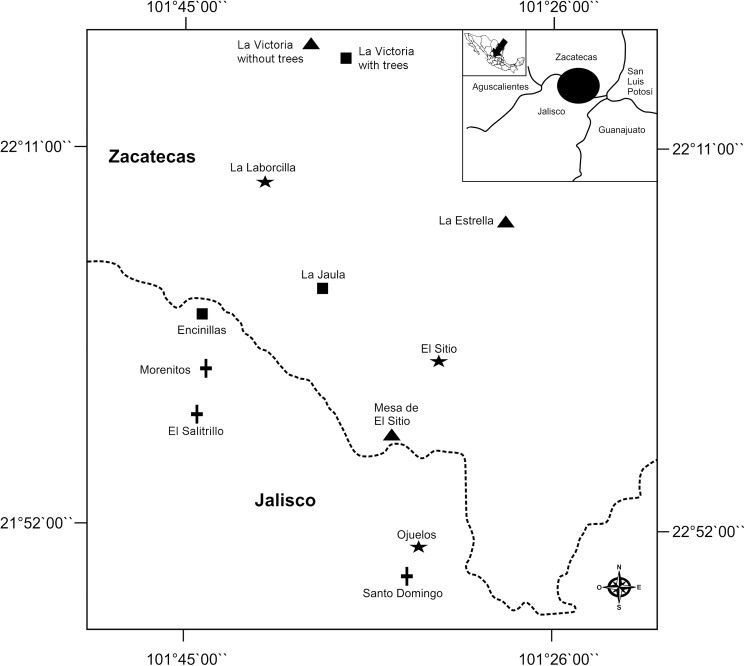
Location of study sites.

This study was carried out in 12 mature (i.e. ≥4 year of established), fruit-oriented nopal orchards (Fig 1). The orchards were grouped according to two factors: Size of the nopal agro-ecosystem patch (regardless of whether it was a single orchard or it was part of a multi-orchard area), and presence or absence of arboreal elements, with three orchards in every combination. Large patches were ≥34 ha, while small patches were ≤9 ha. The arboreal elements (hereafter "trees") were mostly palma china (*Yucca decipiens*), large huizache trees (*Acacia* spp.), and pirul (pepper tree, *Schinus molle*). In each orchard we established a 100 m x 100 m research plot, separated from the edges by at least 20 m. Eleven sites were on ejido (communal) lands, while one was part of a research station. Rodolfo López (Ojuelos), Baltazar Martínez-Gil (El Sitio), Abelardo Contreras (Mesa de El Sitio), the Ejido Encinillas (Encinillas), Beto Camacho (Morenitos), José García (El Salitrillo), Héctor Alvarado (La Jaula), Alfredo Ruíz (La Estrella), Enrique Campos (La Laborcilla), Flavio Esquivel (La Victoria with trees), Mario Álvarez (La Victoria without trees) and the Instituto Nacional de Investigaciones Forestales Agrícolas y Pecuarias and Miguel Luna (Santo Domingo) granted us permission to work in their orchards. Francisco J. Ponce assisted in preparation of Fig 1. All activities were explained to the owner or appropriated authority of each location, and no other activities were carried out.

### Field work and data processing

**Fig 2:**
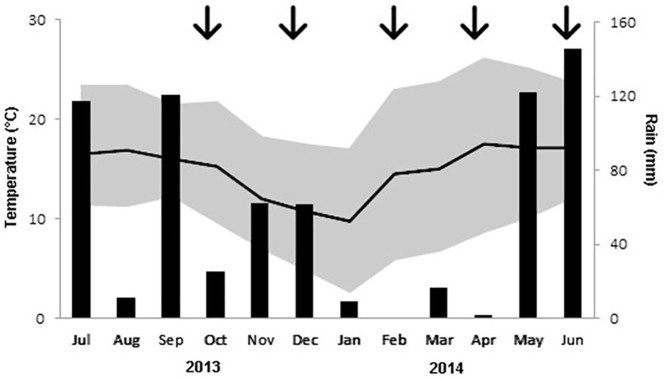
Climatic condition during the study period.

Field work was carried out in five sampling periods (Fig 2): October (30/09-11/10/2013, end of rainy season), December (28/11-13/12/2013, dry cold), February (16-28/02/2014, dry cold), April (13-24/04/2014, dry warm), and June (3-15/06/2014, beginning of rains), under a permit from Dirección General de la Vida Silvestre to EM. Field work was not directed at any species in particular, as it focused on recording species present. No protected species were captured. Two species recorded are protected by Mexican law, Mexican duck (threatened) and Harris hawk (under special protection), but no individuals of either were captured nor otherwise manipulated or disturbed. We did not collect any specimens nor any samples from them. However, the Project was peer-reviewed as part of the funding process and by the graduate committee and personal M.Sc. committee of Melinda Cárdenas, and all instances approved the procedures. The mist nets were used according to standard procedures, checked constantly and any birds captured were released immediately.

#### Bird surveys

We employed two approaches to document the birds using the orchards. The first one was the establishment of three belt transects 30 m wide and 100 m long, in the same direction as the nopal rows. These fixed-width strip transects were surveyed at a pace of 10 m/min thrice before sunset and thrice after sunrise, with 30-min idle periods in between (i.e. the first afternoon survey began 2.5 h before sunset, and the morning surveys ended 2.5 h after sunrise). All birds seen within the transect in connection with the orchard, but not those overflying, were recorded. For each site and period, the highest tally for each species was taken as the best estimator of its abundance. With these maxima, we calculated bird taxonomical diversity values using the exponential of Shannon's entropy index (exp[H’]); [33]).

Our second approach consisted of placing four mist nets (2.6 m high and 12 m long, with 4 shelves and 65 mm mesh) 10 m outside the survey plot, one at each corner. Nets were opened for three hours before sunset and three hours after sunrise, and were checked every 20 min. All birds captured were identified and released unharmed at the spot.

#### Vegetation estimates

Vertical habitat heterogeneity is often the leading intra-habitat variable relevant to birds. To evaluate it we calculated "foliage height diversity" (FHD) using a vertical board, 30 cm wide and 240 cm tall, divided in 25 cm sections, starting 5 cm from the bottom [34]. An observer (MC-G) was placed halfway down each side of the study plot, in the middle of an alley between nopal rows. The board was placed 15 m away from the researcher within the same alley, and 10 m away at 45° (crossing rows of nopal). The percent obstruction from vegetation of each 25-cm section was estimated visually looking horizontally at the board; above 170 cm from the ground, the readings were slanted. The observation points and bearings were the same throughout the study. Shannon diversity values were calculated from square-root transformed percentages of the different sections [34]. Values along alleys and values across them were calculated for each site and period.

We determined ground cover by plants at 600 intercept points along four transects, each one starting 10 m from each corner towards the center of the plot. Points were spaced every 10 cm, and at each we recorded the cover class (or classes, if more than one was intercepted). Classes were: bare ground, litter, herb, shrub, nopal, and tree. Percent cover per class was calculated and square-root transformed.

#### Food abundance

Many of the bird species in the region are granivores or insectivores, and food abundance was calculated through two rough proxies. The first was the placing of four 20 x 20 cm cardboards smeared with car grease, one placed 1 m beyond each plant cover line transect. They were placed 3 h before sunset of one day and retrieved 3 h after sunrise the following morning. In the laboratory, seeds were removed, washed in warm water to remove grease, dried, identified, and counted. We created a seed reference collection by collecting present herbaceous plants with seeds. Seed numbers per plot and period were used as a proxy to seed abundance.

The second proxy focused on arthropods, for which we used a "window trap"; a 60 x 80 cm glass panel placed 100 cm above the ground, with a recipient filled with soapy water underneath [35]. This trap captures arthropods when they hit the glass, fall, and drown. The trap was placed in the center of the study plot 3 h before sunset on an afternoon and retrieved 3 h after sunrise the next morning. All arthropods were collected and preserved in 70% alcohol and identified to taxonomic order.

### Data analysis

We initiated data analysis by exploring them through Principal Components Analyses (PCA). We eliminated all species that appeared only once in our data set. We then performed separate analyses for (1) sites and survey periods, and (2) only sites, with the survey periods pooled. PCAs were carried out in Statistica 7.1.

We then analyzed the data under an information-theoretic framework [36]. We modeled the value of the bird community variables, or the abundance of migratory and of ubiquitous species (those appearing in at least four of the five sampling periods) against groups of environmental variables. The bird community variables analyzed were: species richness, total abundance, species diversity (exp[H’]), abundance of migratory sparrows (lark bunting [*Calamospiza melanocorys*], grasshopper [*Ammodramus savannarum*], lark [*Chondestes grammacus*], vesper [*Pooecetes gramineus*], chipping [*Spizella passerina*] and clay-colored [*S*. *pallida*], due to their high, albeit seasonal presence in the community), general abundance without migratory sparrows, richness of ubiquitous species, overall abundance of ubiquitous species, and abundance of each ubiquitous species. All bird variables were considered to have a Poisson distribution, except for exp(H’), which had a Gaussian distribution (after [37]). Before processing the models, we examined graphically if there was an obvious non-linear relationship between the response and explanatory variables.

Explanatory variables in the models were arranged in four groups: (1) experimental variables (presence / absence of trees, size of patch, survey period), (2) vertical habitat structure (foliage height diversity in its two measures), (3) ground cover (cover of vegetation classes, bare ground, litter), and (4) food (abundance of seeds and of arthropods). Experimental variables were categorical, while the rest of explanatory variables had a Gaussian distribution. We modeled each response variable with all possible combinations of the explanatory variables in each group (8, 4, 57, and 4 models in each group, for each of the 16 response variables).

We modeled all possible combinations within each group in addition of a null model that included only locality. Best models were selected based on their values of the Akaike Information Criterion for small samples (AICc). For parsimony, we selected the simplest model among those being less than about 2.5 AICc units from the one with lowest AICc value. We indicate this in the result tables. When two models were equally simple, by parsimony, we also indicate it in the tables. All modeling was performed using pgirmess and lme4 libraries in R 3.3.1 [38], through RStudio Ver. 0.99.903.

## Results and discussion

### Birds and the effects of the experimental variables

The mist nets were open for 216 h, and trapped 49 individuals of 14 species. All trapped species were recorded with the same or higher numbers at the same time along the fixed transects. Captures were biased to mid-size species flying low over the ground. Given this, mist net data did not help understand the processes involved and we analyzed and present here only the data from the transects.

During the study we counted 998 individuals of 55 different bird species, plus two that could be identified only to genus and could have been a species identified at other times, or a different one (S1 Table). Of all species, 53% were primarily insectivores, 24% granivores, 9% carnivores, 7% nectivores, 3% omnivores, and 3% frugivores. Nine species were ubiquitous: mourning dove (*Zenaida macroura*), Say's phoebe (*Sayornis saya*), cactus wren (*Camphylorhynchus brunneicapillus*), Bewick's wren (*Thryomanes bewickii*), curve-billed thrasher (*Toxostoma curvirostre*), canyon towhee (*Melozone fusca*), black-chinned sparrow (*Spizella atrogularis*), black-throated sparrow (*Amphispiza bilineata*), and house finch (*Haemorhous mexicanus*). Of all species, 50% were present in both large and small patches, 20% were found only in large patches, and 30% only in small ones. Likewise, 51% of all species were found in orchards with and without trees, 34% only on those with trees, and 15% only in those without them.

When the data were arranged by study sites and survey periods, PCA factors 1 and 2 explained 7.52% and 6.91%, respectively, of the total variance. When the data were pooled across survey dates per site, factors 1 and 2 explained 18.07% and 13.33%, respectively. Neither case exhibited an identifiable pattern.

Patch size was an important component only for the abundance of ubiquitous species models, in which orchards in large patches of nopal agro-ecosystems had 9.4±10.07 individuals (average of maximum counts along survey transects), while those in orchards in small patches had 9.13±8.46; and that of black-chinned sparrow which was more than five times more abundant in orchards in small patches than in those in large patches (0.93±1.6 *vs*. 0.17±0.38 sightings per orchard).

When presence / absence of trees was part of the best model, orchards with trees had 49% more species, a 29% higher diversity value, 60% more of ubiquitous bird species, 65% more individuals of ubiquitous species, 3.7 times more cactus wrens, and 2.7 times more Bewick's wrens than orchards without trees (Table 1). Attributes for which "trees / no trees" was part of the best model, but not in the most parsimonious one, were: overall abundance of all birds excluding migratory sparrows, abundance of mourning doves, and abundance of black-chinned sparrows, which benefited from the presence of trees (49%, 2.3 and nearly 5 times higher in orchards with than in those without, respectively) (Table 1).

**Table 1:** Effect of trees in nopal orchards on bird communities and species (+).

Bird attribute or abundance of ubiquitous species	With trees	Without trees
Species richness	7.87±2.40	5.27±2.46
Total abundance	-	-
Species diversity (exp[H'])	6.09±2.19	4.23±1.87
Abundance of migratory sparrows	-	-
General abundance without migratory sparrows	15.17±9.24*	10.2±9.44*
Richness of ubiquitous species	4.53±1.55	2.77±1.50
Abundance of ubiquitous species	11.53±9.44	7.0±8.57
Abundance of mourning dove	3.9±0.08*	0.8±2.20*
Abundance of Say's phoebe	-	-
Abundance of cactus wren	1.23±1.50	0.33±0.80
Abundance of Bewick's wren	0.7±0.79	0.26±0.69
Abundance of curve-billed thrasher	-	-
Abundance of canyon towhee	-	-
Abundance of black-chinned sparrow	0.77±1.43*	0.33±0.92*
Abundance of black-throated sparrow	-	-
Abundance of house finch	-	-

(+) Indicated only in the cases in which it was a variable in the best model (mean ± standard deviation). A dash (-) indicates that the presence / absence of trees was not a factor in the best model of the species, while an asterisk (*) indicates that it was included in the best model, but not in the most parsimonious one and is therefore not deemed relevant.

**Fig 3:**
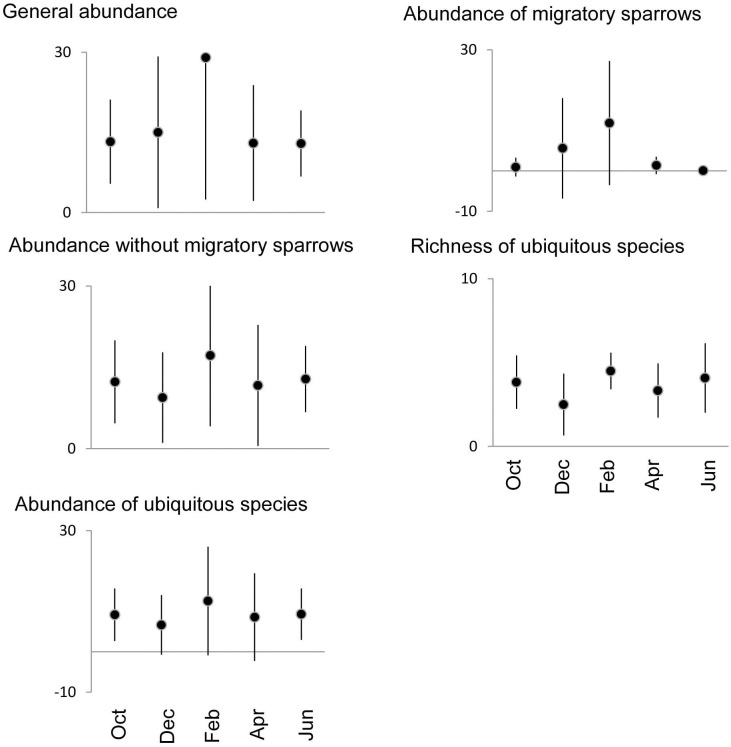
Temporal changes in bird community parameters whose best models included month of survey as an explanatory variable. Mean and standard deviation are displayed.

**Fig 4:**
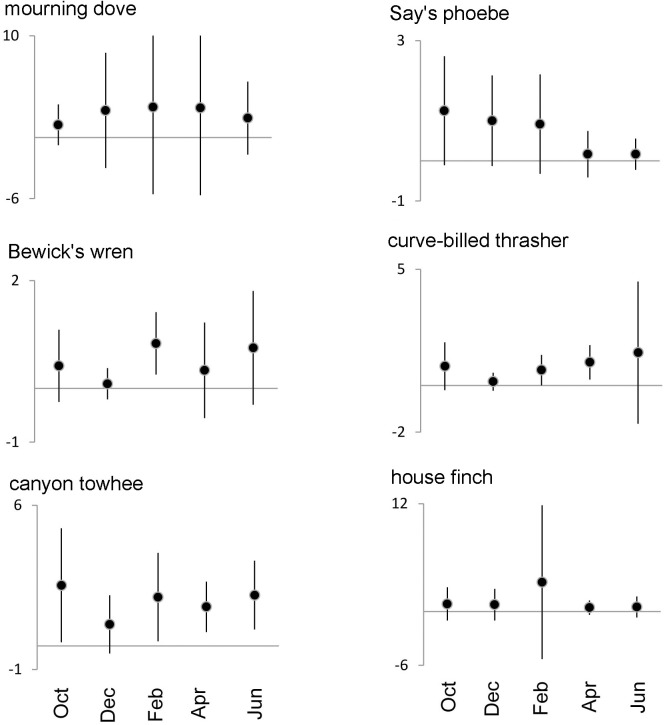
Temporal changes in the abundance of ubiquitous birds whose best models included month of survey as an explanatory variable. Mean and standard deviation are displayed.

Survey period was included in the best models for total abundance, abundance of migratory sparrows, overall abundance without migratory sparrows, richness and abundance of ubiquitous species, and the abundance of mourning dove, Say's phoebe, Bewick's wren, curve-billed thrasher, canyon towhee, and house finch (Figs 3 and 4). Although there was some variation, in most cases February had the highest numbers.

Overall, habitat variables were affected little by the presence of trees and size of the patch, except for: shrub cover was higher, and herb cover and abundance of arthropods were lower in orchards with trees; herb cover and cover by litter were lower, but bare ground and abundance of arthropods were higher in plots in larger patches, although in all cases, differences were rather small and variation high (S2 Table). Orchards with threes had a higher cover by shrubs and by nopales, although in the latter case the difference was small (11.51±0.56% ground cover by nopales in orchards with trees *vs*. 9.46±0.78% in those without).

On the other hand, time of survey influenced several habitat variables: herb cover, bare ground and litter, and production of seeds and arthropods (S3 Table). At the onset of the study the summer rains had already fallen (Fig 2), and herbs were at their maximum ground cover in our study orchards; they matured by December. In February and April the herbs had dried and decayed, transforming patches into those with litter and, in a small proportion, bare ground. In 2014, the rainy season began in May 2014 and, by our June survey, herbs had started to grow.

**Fig 5:**
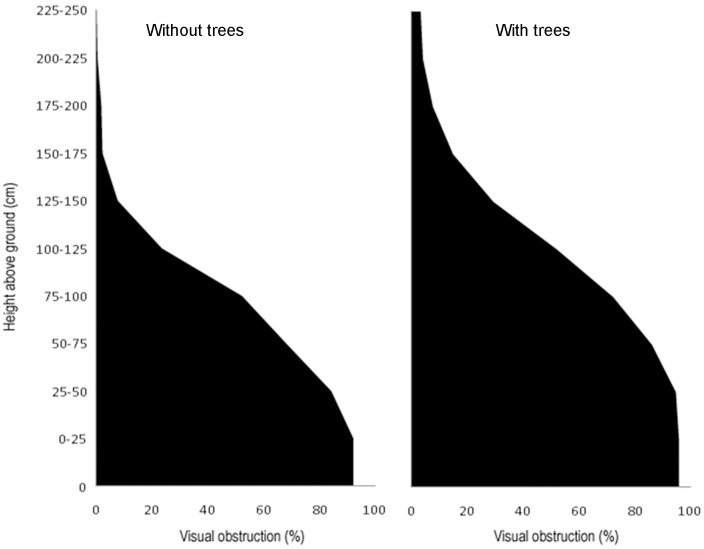
Vertical vegetation profiles in nopal orchards with and without trees.

On the whole, orchards with trees had a more complex vertical profile than orchards without them (Fig 5). Birds, except for the abundance of Say's phoebe and black-chinned sparrow, were influenced by vertical structure of the vegetation at least in one direction: along the alleys (FHD-along), which represents basically herbage, or across nopal rows (FHD-across), which is more a reflection of nopal plants and shrubs (Table 2). Along-alley FHD influenced species diversity and some species in a positive way, but other bird community traits and species negatively, while across-nopal rows FHD was positively associated with 12 out of 16 bird traits and species.

**Table 2:** Relationship between bird community attributes and abundance of ubiquitous species with foliage height diversity (FHD) and abundance of seeds and arthropods (+).

Bird attribute	FHD		Food abundance	
	Along alleys	Across rows	Seeds	Arthropods
Species richness	-	9.09*	-	-
Total abundance	-1.31	35,51	0.084	0.077
Species diversity (exp[H'])	2.39	4.64	-	-
Abundance of migratory sparrows	-4.53	8.38	-0.045	0.073
General abundance without migratory sparrows	-	27.13	-0.04*	-
Richness of ubiquitous species	-	6.29*	-	-
Abundance of ubiquitous species	-	28.83	-0.041	0.016
Abundance of mourning dove	-0.96	-	-	0.007
Abundance of Say's phoebe	-	-	-	-
Abundance of cactus wren	-	1.45*	-	-
Abundance of Bewick's wren	-0.41*	2.04*	-0.015*	-
Abundance of curve-billed thrasher	1.48	-	-	-0.023
Abundance of canyon towhee	1.12*	2.86*	-	-
Abundance of black-chinned sparrow	-	-	-	-
Abundance of black-throated sparrow	-0.16*	-	-	-
Abundance of house finch	0.64*	9.53*	0.033	0.049

(+) Values given are of the slope of a regression. An asterisk (*) denotes that the variable indicated appeared in the best model, but that, by parsimony, the null or another, simpler model was as good and should be considered the best one.

Except for species richness and richness of ubiquitous species, and the abundance of black-throated sparrows, bird attributes where explained best by a model including at least one ground cover variable (Table 3). However, in most cases, the shallow slope of the relationship indicates that the effects were small, and in case of the canyon towhee, the best model was not any better than the null model.

**Table 3:** Relationship between bird community attributes and abundance of ubiquitous species and ground cover (+).

Bird attribute or abundance of ubiquitous species	Tree cover	Shrub cover	Herb cover	Nopal cover	Bare ground	Litter
Species richness	-	-0.1	-	-	-	-
Total abundance	-0.55	-0.46	-0.09	-	-0.01	-
Species diversity (exp[H'])	-	-	-	-	-	-
Abundance of migratory sparrows	-0.56	-0.15	-0.05	-	-0.03	0.03
General abundance without migratory sparrows	0.02	-0.31	-	-	0.02*	0.07
Richness of ubiquitous species	-	-	-	-	-	-
Abundance of ubiquitous species	0.09*	-0.3*	-0.003	0.2*	-0.002	0.05
Abundance of mourning dove	-0.18	-0.02	-0.01	0.28	0.04	-0.005
Abundance of Say's phoebe	-	-0.05*	-	-	-	-0.02
Abundance of cactus wren	0.3	-	-	0.05*	-	-
Abundance of Bewick's wren	-	-	-	0.06	-	-
Abundance of curve-billed thrasher	-	-	-	0.06	-0.02	0.02
Abundance of canyon towhee		-0.09*				
Abundance of black-chinned sparrow	0.2	0.005	0.003			0.01
Abundance of black-throated sparrow						
Abundance of house finch	-0.14*	-0.12				0.04

(+)Values given are of the slope of a regression. An asterisk (*) denotes that the variable indicated appeared in the best model, but that, by parsimony, the next model, containing only the other variables, or the null model if no other variables are indicated, is as good and should be considered the best one. Values rounded to two digits, except when the resulting value would have been 0.

### Effect of abundance of food on the birds

Seed traps collected 441 seeds of 17 species. Of these, 16 species were part of our reference collection (which included 30 species of 9 families), 14 grass and two species of Asteraceae. There were no clear effects of presence or absence of trees, nor of patch size, on seed abundance (S1 Table), but seed production followed a clear chronological pattern. Following the maturing of the herbs, the December seed catch was 4.4 times higher than the average of the other four surveys, which were very uniform. Subsequently, with the decay of the herbs, many more seeds probably reached the ground before our February survey.

Throughout the study the window trap collected 762 arthropods of at least 242 types, including morphospecies and gender classes. The most represented were flies, mosquitoes, and gnats (Diptera; 34–77% per period of all identifiable individuals, 81 types), followed by beetles (Coleoptera; 8–29%, 27 types), true bugs, and bees, ants and wasps (Hemiptera and Hymenoptera; 3–21, 1–7%, respectively, 32 types each). Other groups were less represented. Arthropods were present throughout the study but peaked in February, although with a low peak. Models including the abundance of seeds and or arthropods, were better than the null model in explaining bird attributes in half of the cases, but in all with a shallow slope (Table 2).

Most of the birds detected in this study have been found before in nopal orchards of the region [12]. However, 6 of 55 species were recorded in the orchards for the first time, although they are common in the region, showing that our knowledge of this system is still incomplete. The nine ubiquitous species are all common in different habitats of the region. Five of them have been reported as consumers of tunas [23], and we have confirmed seven of them to nest in nopal orchards [12]. Overall, the birds assemblages documented in this study confirm that, unlike other agro-ecosystems, nopal orchards are not purely alien, artificial habitats, but they blend with shrub communities of the regional landscape, largely because their structural attributes resemble that of natural xerophytic shrublands.

Of the factors that influence bird communities, patch size has been touted as one of the major drivers of biodiversity in fragmented landscapes [31, 39]. However, in our study it had little or no influence bird communities in any detectable way, except for black-chinned sparrows, which were more abundant in small patches. This might due to the highly heterogeneous nature of the local landscape [11], in which even plowed plots have near-natural shrubby fencerows, and grasslands have shrubs as a result of overgrazing. At the same time, the local landscape is a finely grained patchwork of sites in which even orchards that do not form part of large patches are not too far from other orchards or shrubby patches and of patches of "stepping-stone habitat,” allowing unrestricted movement of birds between orchards. Thus, from most birds' point of view, the orchards are all but isolated and unreachable.

Lack of an effect of patch size on bird communities supports the idea that small habitat patches of natural and seminatural habitat provide refuge for wildlife. Also, nopal orchards, despite being an agro-ecosystem, structurally resemble natural shrublands. Their major component, cultivated nopal, has derived from species native to the region [12, 22], and so they can be considered as seminatural habitats. The orchards providing high quality habitat and being at least ~6 ha appear to have been adequate to sustain local populations of the most sedentary bird species.

Clearly, the presence of trees inside the orchards was the major overall feature in creating a favorable habitat and attracting birds to them, benefiting birds in general, as well as some selected species. Given their relative small cover and biomass, scattered trees in many habitats have a disproportionately large contribution to ecosystem functioning [4, 40–41]. It has been argued that trees allow the presence of more species as they offer resources that reduce interspecific competition [4]. They also offer resources not provided by other components of the orchards. For example, phainopeplas (*Phainopepla nitens*) foraged on insects flying around tree canopies. We cannot assert that the differences between orchards with and without trees were caused solely by the trees as the prior had also slightly higher cover by shrubs and by nopales. This was reflected as a more complex vertical profile across nopal rows in the orchards with trees. Although the difference in profiles is not dramatic, it is caused by leafy plants which are structurally different from nopal and, therefore, have an effect disproportionate to their actual cover or biomass. The higher abundance of cacti and Bewick's wrens in orchards with trees probably resulted from their association with the higher presence of shrubs and nopales, more than with the trees themselves. Cactus wrens do indeed use nopales in orchards for nesting.

Complex vegetation provides better cover to hide from predators, and also more microhabitats that satisfy the requirements of different bird species. In the fruit-oriented nopal orchards of our study area, the particular structure of the crop itself imparts a highly heterogeneous structure to the system, which is augmented by the presence of other shrubs, spontaneous native nopal plants of several species, herbs and grasses and, often, trees, either within the farmed area or at its edges [12]. Thus nopal orchards conform with the well-established positive relationship between heterogeneity and biodiversity in agro-ecosystems [7].

The other major driver of bird communities in our study was survey time, reflecting a strong seasonality. This was well evidenced by more species and more individuals in February, which was caused largely by the arrival of migratory sparrows. Herbaceous vegetation was at its maximum in September as a consequence of the summer rains, and matured until the early winter when seeds falling on the ground increased four-fold. After December, the dry herbs decayed, and large openings (in many cases ≥100m^2^) had formed by February. The combination of abundant seed and open patches, coinciding with the migration chronology of the sparrows, provided optimal conditions for their feeding in February.

Several species that use orchards have conservation problems throughout their range. Two, black-chinned sparrow and scaled quail (*Callipepla squamata*) are on PIF´s “Yellow Watch List—Species with population declines and moderate to high threats", and have respectively experienced global population reductions of 61 and 67% during the past 44 years. The black-chinned sparrow had >5 times higher numbers in small patches, and its numbers were more than double in orchards with trees than in those without. This species also nests in shrubs and small trees in nopal orchards and in shrublands [12]. This resident, ubiquitous, appealing species would be a prime subject for research and conservation-oriented management, but also is charismatic enough to be used as flagship species in conservation campaigns. The scaled quail, despite being a grassland species, in our area is more common in orchards, even breeding in some of them [12]. It did not exhibit any preference for patch size or tree presence.

Grassland birds are under strong conservation pressures throughout the continent. In our case, all migratory sparrows have exhibited population reductions between 1966 and the present, ranging from 35 to 93% of their populations [42]. The most serious declines have been suffered by grasshopper sparrows and by lark buntings (75 and 93% population reductions since 1966, respectively), and they are considered “common birds in steep decline” (PIF; [42]). Lark buntings, and vesper and chipping sparrows preferred orchards with trees, but the clay-colored sparrows preferred those without them. Also, whereas lark buntings preferred large orchards, chipping and clay-colored sparrows preferred small ones. Grasshopper sparrows were not abundant in our study. Thus, to benefit the guild of migratory sparrows, a patchwork of sites including large and small orchard patches and orchards with and without trees would be needed. However to benefit the species apparently most in need, the lark bunting, orchards in large patches, with trees, would be the best choice.

Another “common bird in steep decline” is the cactus wren. This is one of the signature birds of the orchards. It is a permanent resident of the orchards, where they find food, shelter, and nesting habitat. In our study they were almost four times more abundant in orchards with trees than in those without them, and keeping orchard habitat suitable for them could be a management objective. Our findings are a step forward in understanding how to tailor orchards for conservation. Also, they might suggest future venues of research.

The idea that traditional agro-ecosystems are intrinsically better for biodiversity than other systems has been around for some time [43, 44]. However, although some traditional systems truly conserve local biodiversity, this is not always straightforward. In our study area, most cropland is devoted to the centuries-old traditional growing of corn and beans, with native herbs being a common component of them. However, they are not bird-rich systems [12]. In contrast, fruit-oriented nopal orchards, which were first established in the 1950s and, hence cannot be considered truly "traditional", although they are based on varieties of native species, hold rich bird communities and offer a conservation alternative in the Llanos de Ojuelos and beyond.

Nopal orchards, although having a basic planting pattern, differ in whether they are isolated or form part of large areas with the same crop, and in whether they have trees, among other characteristics. This results in a variety of orchard patches of different size and internal vertical structure. Whether an orchard has trees and other plants or not is a decision of the landowner, more than given by the technification level of the orchard. By the same token, wintering sparrows can be easily benefited by allowing herbs and grass to grow inside the orchards with the summer rains, and allowing them to seed. If for any reason no clearings are present in the orchard in January, they can be easily created.

## Conclusions

Based on the work here presented, we conclude that:

1. The major driver of bird communities in nopal orchards, by far, was the presence of trees within the orchard, which affected positively most bird community attributes measured.
2. The second major driver of bird communities was seasonality. Although with some variation, the most common pattern was a February peak in the value of the attributes measured. The February presence of migratory sparrows was favored by large quantities of seed in the ground, in open patches, but near escape cover and shelter.
3. Nopal orchard patch size was irrelevant in structuring bird communities, and had a minor effect on only the abundance of all ubiquitous species together. However, small patches benefited black-chinned sparrows, a species with conservation concerns.
4. These orchards provide adequate habitat and food resources for different functional bird guilds, especially insectivore species, but also granivores, omnivores, nectivores, and some birds of prey.
5. The major attributes that made orchards important for birds in our study: trees, shrubs, herbs, seeds, and open patches, can be managed easily to maintain native biodiversity in this highly anthropized region with urgent needs of finding a convergence between production of goods and biological conservation.

## Supporting information

S1 Table List of bird species recorded in fruit-oriented nopal orchards while studying the effects upon them by presence / absence of trees and size of the patch of orchard habitat, in the Llanos de Ojuelos, Jalisco and Zacatecas, Mexico. 2013–2014.
(PDF)

S2 Table Effect of the presence or not of trees and the size of the patch in which the orchard was on explicative variables of birds in the Llanos de Ojuelos, Jalisco and Zacatecas, México. 2013–2014. Mean and standard deviation are presented.
(PDF)

S3 Table Effect of survey period on explicative variables of birds in the Llanos de Ojuelos, Jalisco and Zacatecas, México. 2013–2014 (mean ± standard deviation).
(PDF)
