# Supplementary material for: Biodiversity conservation in an anthropized landscape: Trees, not patch size drive, bird community composition in a low-input agro-ecosystem
Source: PLoS One. 2017 Jul 7;12(7):e0179438. doi: 10.1371/journal.pone.0179438 (PMC5501394; doi:10.1371/journal.pone.0179438)
Supplement: S1 Table — 2013–2014. (PDF) [file pone.0179438.s001.pdf]

S1 Table. List of bird species recorded in fruit-oriented nopal orchards while studying the effects upon them by presence / absence of trees and size of the patch of orchard habitat, in the Llanos de Ojuelos, Jalisco and Zacatecas, Mexico. 2013-2014.

Anatidae	*Anas plathyrrynchus*
Odontophoridae	*Callipepla squamata*
Accipitridae	*Elanus leucurus*
	Circus cyaneus
	Parabuteo unicintus
	Buteo jamaicensis
Falconidae	*Falco sparverius*
Columbidae	*Zenaida asiática*
	Zenaida macroura
	Columbina inca
Cuculidae	*Geococcyx californianus*
Caprimulgidae	*Chordeiles acutipennis*
Trochilidae	*Hylocharis leucotis*
	Archilochus sp.
	Selasphorus platycercus
Picidae	*Melanerpes aurifrons*
	Picoides scalaris
	Colaptes auratus
Tyrannidae	*Contopus sordidulus*
	Empidonax sp.
	Sayornis saya
	Myarchus cinerascens
	Tyrannus vociferans
Laniidae	*Lanius ludovicianus*
Vireonidae	*Vireo huttoni*
Corvidae	*Aphelocoma californica*
	Corvus cryptoleucus
Alaudidae	*Eremophila alpestris*
Hirundinidae	*Hirundo rustica*
Remizidae	*Auriparus flaviceps*
Troglodytidae	*Camphylorhynchus brunneicapillus*
	Thryomanes bewickii
Polioptilidae	*Polioptila caerulea*
Mimidae	*Mimus polyglottos*
	Toxostoma curvirostre

Ptilogonatidae	*Phaenopepla nitens*
Parulidae	*Setophaga coronata*
	Cardellina pusilla
Emberizidae	*Melozone fusca*
	Spizella sp.
	Spizella atrogularis
	Spizella pallida
	Spizella passerina
	Amphispiza bilineata
	Calamospiza melanocorys
	Pooecetes gramineus
	Chondestes grammacus
	Ammodramus savannarun
Cardinalidae	*Pheuticus melanocephalus*
	Passerina caerulea
Icteridae	*Sturnella* sp.
	Agelaius phoenicius
	Molothrus aeneus
	Icterus sp.
	Icterus parisorum
Fringillidae	*Haemorhous mexicanus*
	Spinus saltria
