# Supplementary material for: Biodiversity conservation in an anthropized landscape: Trees, not patch size drive, bird community composition in a low-input agro-ecosystem
Source: PLoS One. 2017 Jul 7;12(7):e0179438. doi: 10.1371/journal.pone.0179438 (PMC5501394; doi:10.1371/journal.pone.0179438)
Supplement: S2 Table — 2013–2014. Mean and standard deviation are presented. (PDF) [file pone.0179438.s002.pdf]

S2 Table. Effect of the presence or not of trees and the size of the patch in which the orchard was on explicative variables of birds in the Llanos de Ojuelos, Jalisco and Zacatecas, México. 2013-2014. Mean and standard deviation are presented.

	Trees		Size	
	Yes	No	Large	Small
FHD-along	1.08±0.44	0.67±0.37	0.74±0.54	1.01±0.3
FHD-across	1.77±0.23	1.54±1.89	1.65±0.23	1.66±0.25
Tree cover	1.21±2.06	0	0.43±1.01	0.78±1.98
Shrub cover	4.22±4.87	1.45±3.36	3.17±5.14	2.5±3.51
Herb cover	27.68±19.9	33.93±27	24.22±20	37.4±25.5
Nopal cover	11.51±3.11	9.47±4.29	11.58±3.65	9.4±3.8
Bare ground	25.65±12.3	22.65±24.7	34.88±21.6	13.42±8.06
Litter	29.43±19.1	29.92±23.8	25.23±17.4	34.12±24.3
Seeds	7.7±9.57	7±12	6.03±9.57	8.67±11.8
Arthropods	10.47±12.9	15.2±13.1	14.73±12.9	10.93±9.54
