# Supplementary material for: Biodiversity conservation in an anthropized landscape: Trees, not patch size drive, bird community composition in a low-input agro-ecosystem
Source: PLoS One. 2017 Jul 7;12(7):e0179438. doi: 10.1371/journal.pone.0179438 (PMC5501394; doi:10.1371/journal.pone.0179438)
Supplement: S3 Table — 2013–2014 (mean ± standard deviation). (PDF) [file pone.0179438.s003.pdf]

S3 Table. Effect of survey period on explicative variables of birds in the Llanos de Ojuelos, Jalisco and Zacatecas, México. 2013-2014 (mean ± standard deviation).

	2013		2014		
Variable	Oct	Dec	Feb	Apr	Jun
FHD-along	0.98±0.38	0.88±0.39	0.74±0.52	0.91±0.5	0.88±0.51
FHD-across	1.72±0.26	1.58±1.74	1.59±0.25	1.62±0.2	1.75±0.26
Tree cover	0.96±2.32	0.25±0.87	0.42±1	0.61±1.5	0.79±1.85
Shrub cover	2.76±6.63	3.58±4.66	1.46±2.17	3.5±3.99	2.87±3.66
Herb cover	47.38±20.98	47.54±22.79	11.04±8.03	9.17±99	38.92±17.17
Nopal cover	10.42±3.1	10±4.34	9.71±3.43	11.71±3.71	10.62±4.81
Bare ground	20.21±18	19.12±20.12	27.25±21.29	26.83±20.07	27.33±19.14
Litter	18.29±11.21	19±11.13	43.87±23.13	48±21.18	19.21±15.8
Seeds	4.83±10.86	19.58±13.36	4.42±4.93	6.42±8.14	1.5±3.8
Arthropods	11.08±5.73	13±13.44	18.33±10.87	11.17±8.3	10.58±15.98
